# Supplementary material for: Shotgun metagenomic analysis of saliva microbiome suggests Mogibacterium as a factor associated with chronic bacterial osteomyelitis
Source: PLoS One. 2024 May 6;19(5):e0302569. doi: 10.1371/journal.pone.0302569 (PMC11073694; doi:10.1371/journal.pone.0302569)
Supplement: S1 Fig — Genera showing a difference of more than 10% are highlighted in this plot. (PPTX) [file pone.0302569.s001.pptx]

## Slide 1
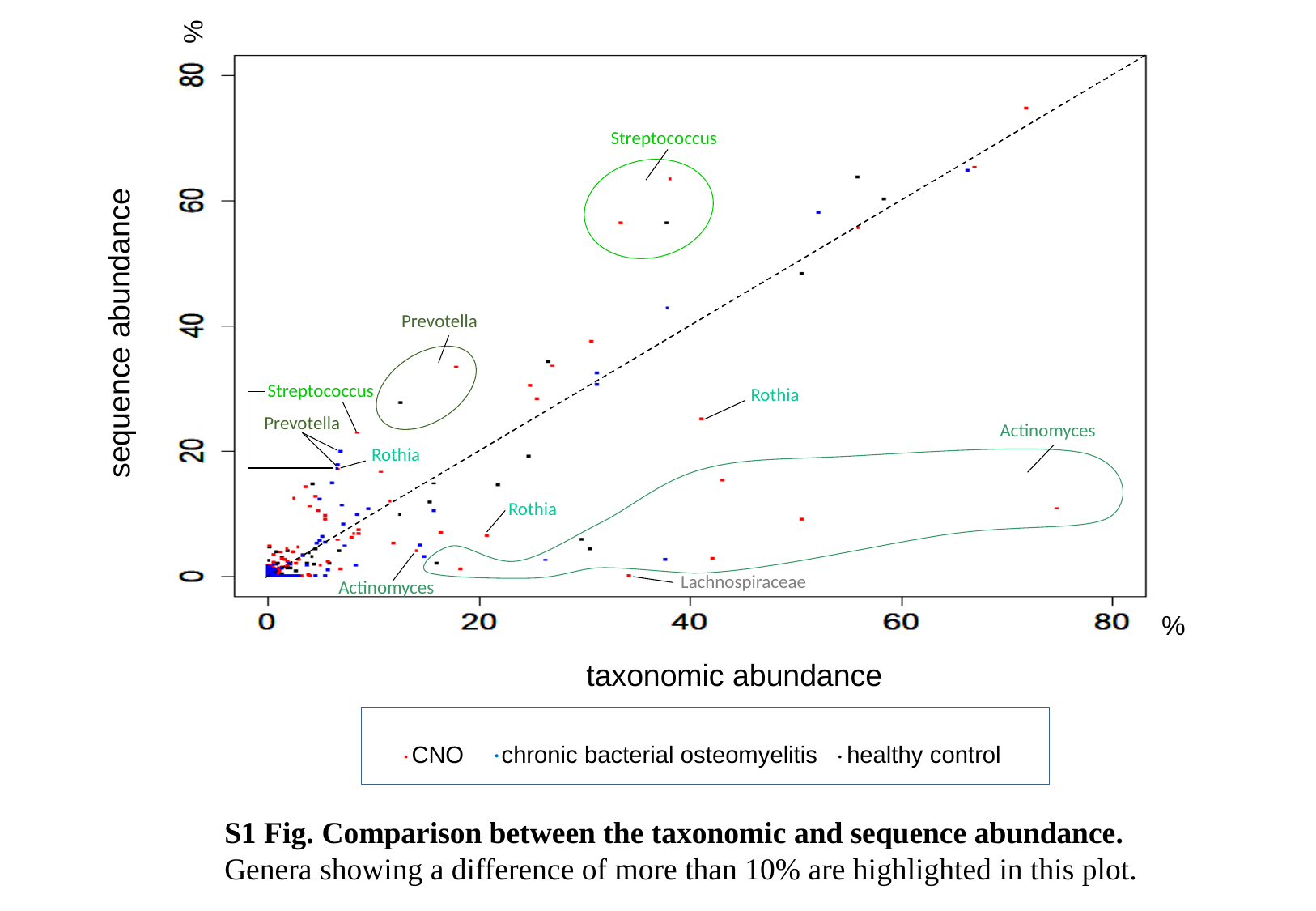

%
Streptococcus
Prevotella
sequence abundance
Streptococcus
Rothia
Prevotella
Actinomyces
Rothia
Rothia
Lachnospiraceae
Actinomyces
%
taxonomic abundance
● CNO ● chronic bacterial osteomyelitis ● healthy control
S1 Fig. Comparison between the taxonomic and sequence abundance.
Genera showing a difference of more than 10% are highlighted in this plot.
