# Supplementary material for: Shotgun metagenomic analysis of saliva microbiome suggests Mogibacterium as a factor associated with chronic bacterial osteomyelitis
Source: PLoS One. 2024 May 6;19(5):e0302569. doi: 10.1371/journal.pone.0302569 (PMC11073694; doi:10.1371/journal.pone.0302569)
Supplement: Attachment — Submitted filename: response.docx [file pone.0302569.s005.docx]

**Editor:** I have completed my evaluation of your manuscript, which has been reviewed by two highly qualified reviewers all of whom agree it is worth to be published in PLOS ONE. Nevertheless, they have suggested some changes that will help to improve the paper.

Therefore, I invite you to resubmit your manuscript after addressing the reviewers’ comments below. When revising your manuscript, please consider all issues mentioned in the reviewers' comments carefully: please, outline every change made in response to their comments and provide suitable rebuttals for any comments not addressed. Please, note that your revised submission may need to be re-reviewed.

PLOS ONE values your contribution and I look forward to receiving your revised manuscript.

Response: Thank you for your positive evaluation and allowing us to resubmit the manuscript after revisions. We have addressed all the reviewer’s comments and have responded, as presented below.

**Reviewer #1:** The authors have presented a well-written, clear and concise paper to show the results of a study to investigate the salivary bacterial composition of people with osteomyelitis. The abstract and introduction are logical and complete, the materials and methods present the sampling and data creation well, and the results and discussion bring the work together and explain the conclusions well. My only comment is the number of samples that were taken, being n=10 and n=5 respectively for different types of osteomyelitis. It is a small sample set, but I do understand that this is an exploratory and descriptive study. Are there other datasets to compare these results with?

Response: Thank you for acknowledging the significance of our study. However, additional datasets of salivary shotgun metagenomics obtained for different types of osteomyelitis are unavailable. Thank you again for your understanding that this is an exploratory and descriptive study with a small sample size, as indicated in the Conclusion section.

I was pleased to see the depth of sequencing- in my experience the hardest consequence of salivary studies is to gain enough bacterial reads above the human reads. A final question is what the overall bacterial abundance is in real terms and how this relates to the healthy controls. Could increase in relative abundance of Mogibacterium be a consequence of a proliferating bacterial counts in the saliva?

Response: Thank you for this valuable comment. The overall bacterial absolute abundance in real terms cannot be obtained from the shotgun metagenome sequencing data that are compositional, meaning that sequencing only provides information on the relative abundance of bacterial taxa and each taxon’s observed abundance depends on the observed abundances of all other taxa (as indicated in Nearing et al. (2022), *Nature Communications*, reference [39].

In short, a complete descriptive paper that will likely stimulate understandings of the progression of disease.

Response: We appreciate your positive and encouraging comment.

**Reviewer #2:** Review of the article titled Shotgun metagenomics comparing taxonomic and sequence abundances identifies Mogibacterium associated with chronic bacterial osteomyelitis in the saliva microbiome

Summary

The authors of this article clearly display the signs of expertise and competence in their field. The article is well written and the main points are clearly discussed with understandable and elaborate language. Based on their published literature they are well entrenched in this research field, which is clearly shown from their confidence in handling their data and discussing the results. I believe that the findings of this article are appropriate for publication and they will be a valuable addition in the oral microbiome research. I would like them however to address some of my concerns first, mainly about the statistical analysis that they employed.

Major revisions

My biggest problem with the article is the data analysis in the Taxonomic profiling identifies microbes associated with chronic bacterial osteomyelitis section. Seemingly the “taxonomic abundance” data that you publish in this article (Table S2) is not exactly the same as the data that were used in your statistical analyses that you discuss. I take this from your statement in Line 228-229 in parenthesis and from the fact that I tried to replicate your analyses using Table S2 and I obtained substantially different results.

I performed pairwise Wilcoxon tests between the patient groups across all taxa which yielded more than 4 significant differences with the given 0.05 significance threshold or none with a Bonferroni corrected threshold. I also tried to replicate your boxplots from Figure 1 but I found interesting differences (the position of the median line, position of your data points between the 25th and 75th percentiles), which – for me – suggest that there was an underlying distribution (differing from the data published in Table S2) that was used to create these boxplots.

The reason for these discrepancies are not clear to me. If there is a difference between the employed data in the analyses and the published data in Table S2 then this difference should be strongly emphasized in the text.

Response: Thank you for providing such detailed comments and the R script. Regarding the median line, we have confirmed that it is the same (indicating median value 2.2) in Fig 1b and the figure created by the reviewer’s R script, as shown in the comparison below (after changing the y-axis scale interval from 5 to 2 for clarity). The comparison also shows that the data points are the same between the 25th and 75th percentiles (shown as black dots in the middle boxes). Although the 75^th^ percentile is indeed different (11.4 in Fig 1b and 8.5 in the figure created by reviewer’s R script), we confirmed that it is due to a difference in the quantile algorithm between JMP and R: the 75^th^ percentile in Fig 1b (11.4) calculated by JMP is obtained using the 6^th^ type quantile algorithm implemented in R, whereas that in the figure by the reviewer’s R script (8.5) is obtained using the 7^th^ type quantile algorithm, which is the default in R.

Regarding the pairwise Wilcoxon tests, they were overall consistent with the present study: the four taxa showing a significant difference in both the CNO and chronic bacterial osteomyelitis groups compared to the control group included *Abiotrophia* and *Lautropia* (shown in Figure 1a) and *Cardiobacterium* and *Aggregatibacter*, which showed slightly larger P_FDR_ values than 0.05 (0.060 and 0.078, respectively) in the present study. The abundance of *Mogibacterium* showed a significant difference in the chronic bacterial osteomyelitis group compared to the control group but not in the CNO group; thus, it was not included among the four taxa.

I would also like you to include a more detailed description of the data handling steps mentioned in Line 229 (“using a model with default normalization and transformation implemented in MaAsLin2”) in the Methods section, as this would explain why you would get different results from the raw sequence data.

Response: According to your suggestion, we have added the following to the Methods: “According to previous studies [38, 39], we specified the total sum scaling (TSS) normalization (to ensure that the profile values ranged from 0 to 1) and arcsine square-root transformation (AST) (to stabilize the variance and improve parametric estimation models in the presence of violated data assumptions, such as normality and homoscedasticity) [37].” (Lines 216–219) We have also confirmed that the arcsine square-root transformation (AST) is conducted using the following R code "sign(x) * asin(sqrt(abs(x)))", and the taxonomic abundance of *Mogibacterium* (normalized to be from 0 to 1) before and after the transformation is distributed as shown below (left and right, respectively). The transformation seems to make the distribution less skewed.

before AST	after AST

If, however, I should be able to replicate your results from the available data table (Table S2) alone, then please explain to me why I obtained different results from the ones you discussed in this section. For clarity I include my R script (verification.R) for you to check the validity of my claims.

Response: We hope that our above response is sufficient. Again, thank you for providing the R script.

I had another problem specifically with the results concerning the Mogibacterium comparison. In my R script I also try to show you that assuming a simplistic normal distribution, 2 of your samples seems highly likely to be outliers. I don’t want to assume that this type of data is necessarily normally distributed, but this should still indicate that you should be careful interpreting these results. I see that you touch on this topic in Line 407-408, but I would like you to also include a short disclaimer in this section as well, that you are indeed aware of the low sample size of the chronic bacterial osteomyelitis group and the possibility of some samples to be outliers.

Response: We appreciate your comment and agree with it. The illustration of the distribution above shows that the two samples at the right seem to be outliers, even after the arcsine square-root transformation (AST). Therefore, we added the following to the Discussion: “However, the sample size of the present study was small (N=5 in the chronic bacterial osteomyelitis group). Although all five patients with chronic bacterial osteomyelitis showed a greater taxonomic abundance of *Mogibacterium* than the five healthy controls, the abundance values for two of these patients appeared to be outliers that were only distinct relative to those of CNO patients (Fig 1b). Further studies should be performed with additional data on chronic bacterial osteomyelitis patients to explore the external validity of the study and determine the frequency of such outliers, which may represent a potential characteristic of chronic bacterial osteomyelitis. (Lines 390–397)

Minor revisions

Title – I would suggest a modified title “Shotgun metagenomic analysis of saliva microbiome suggests Mogibacterium as a factor associated with chronic bacterial osteomyelitis” or something along this line. As I don’t think your discussion of taxonomic and sequence abundance comparison is that central to this articles message.

Response: We have modified the title accordingly.

I also suggested to change the “identify” part as you yourself describe these results as preliminary in the Conclusion section (Line 407-408).

Response: We have changed the term “identify” to “suggest”, “find”, or “reveal potential” throughout the text.

Line 39 “previous study” – Please include a reference here.

Response: This term corresponds to “A previous meta-analysis conducted in 2021 [37]” at the beginning of “Comparison with a previously reported core saliva microbiome genera” section in Results. Because line 39 is in Abstract, we did not add a reference there; however, we replaced “previous study” with “previous meta-analysis” so that the information is clearly consistent with the sentence in the Results.

Line 168-170 – “we randomly selected the same number of read pairs [..] using BBmap.” – If this is the standard method when account for differences in sequence coverage than please include a reference here where they discuss the reason behind this method. If this is a new approach than please include a description here about the validity of this method.

Response: We have added the following reference for Carr et al. (2020), which states the following: “To account for the differences in read depths across different data sets, the samples were subsampled to the same number of reads across cohorts”.

Carr VR, Witherden EA, Lee S, Shoaie S, Mullany P, Proctor GB, et al. Abundance and diversity of resistomes differ between healthy human oral cavities and gut. Nat Commun. 2020;11(1):693.

Moreover, I think a reference is missing here for BBmap.

Response: We have added a reference as follows: “BBmap, which is included in the BBTools software package [25]”. (Lines 169–170)

25.	Bushnell B. BBTools software package. 2014. Available from: http://bbtools.jgi.doe.gov.

Line 176-177 Which program was used for the concatenation?

Response: We have updated the sentence as follows: “we concatenated the read pairs into a single input file in advance using BBmap while subsampling the reads [25]”. (Lines 176–177)

Line 186-187 “Contig Annotation Tool (CAT) [30]” – I think a reference is missing from here, because the Nakazawa et al. 2002 article is referenced here which clearly belong to Line 196 where it is given appropriately. Please insert the proper reference (this would be von Meijenfeldt et al. 2019, https://doi.org/10.1186/s13059-019-1817-x ?) and number the rest of the references accordingly.

Response: Thank you for pointing out this issue. We have inserted the proper reference, which is indeed Meijenfeldt et al. (2019).

Line 194-195 Mogibacterium is not mentioned prior to this statement, maybe a short introduction should be included to justify why this specific taxon was selected for the phylogenetic analysis.

Response: We have added the following to the beginning of the sentence: “Because we focus on *Mogibacterium* as a characteristic genus associated with chronic bacterial osteomyelitis in the Results section.” (Lines 195–196)

Line 202-203 Where can I find this heatmap, please reference this statement. If the picture/table is not included in the article, why do you mention it?

Response: We have removed “as a heatmap” as suggested.

Line 209-210 I think it would be appropriate to reference both JMP Pro and R software as well in the Reference list to clarify the availability of these softwares.

Response: We have added references for both JMP Pro and R.

Line 253-256 “were highly correlated (Spearman’s correlation coefficient: 0.85) […]” – the other groups seemed also highly correlated, I am not sure whether this difference is high enough to justify discussion

Response: We have modified the sentence as follows: “Overall, among the 74 bacterial genera, the taxonomic profiles were highly correlated between the CNO and chronic bacterial osteomyelitis groups, healthy control and CNO groups, and healthy control and chronic bacterial osteomyelitis groups (Spearman’s correlation coefficient: 0.85, 0.72, and 0.77, respectively).” (Lines 262–265)

Line 284-285 “The blue line indicates the significant enrichment […]” – I cannot see a blue line on Figure 2. Please clarify the meaning of the sentence.

Response: We have removed the sentence because the blue line was removed before our initial submission.

Line 393-395 “[…] using multiple reference genomes across species, […]” – This approach is first mentioned here. Please include a more detailed description of the validity of this method compared to others in the Method section.

Response: We have removed “using multiple reference genomes across species” because it was only included to show that the genus included multiple genomes.

TableS2 The heading of the first column describes sampleIDs, the table however seemingly contains genus names. If it was the intended nomenclature, please clarify why you chose “sampleID”, otherwise correct the header.

Response: We have corrected the text in the heading to “Genus.”

Spelling errors

Line 43 “level” – levels

Response: We have fixed this error.

Line 44 “in a chronic” – in the chronic

Response: We have fixed this error.

Line 45-47 “this is the first study…” – this seems like a strong statement, maybe it would be prudent to lighten it

Response: We have modified the text as follows: “our study presents a comparative analysis …” (Line 46)

Line 81 “chronic specific inflammation” – I don’t think chronic specific is an appropriate term in English. Maybe “CNO is characterized by markers specific to chronic inflammation,” or something along this line.

Response: We have modified the text as follows: “CNO is an inflammatory disorder.” (Line 83)

Line 96 “For instance, a” – an

Line 122 “from previous out study” – from our previous study

Line 133 “techniques have employed” – are employed

Response: We have fixed these errors.

Line 148-149 “A total of 15 patients […] with CNO in Japan”. – “A total of 15 Japanese patients were […]”; or “A total of 15 patients were chosen from Japan for this study […]”. The original statement reads like only the 10 CNO patients are from Japan.

Response: We have modified the sentence as follows: “In total, 15 Japanese patients were prospectively enrolled in this study, including 5 with chronic bacterial osteomyelitis and 10 with CNO.” (Lines 149–150)

Line 160 “data of the five healthy control” – data of five healthy controls

Line 167 “Afte” – After

Line 338 “which may may include” – which may include

Response: We have fixed these errors.

Questions

Line 327-328 – I am not sure about the origin of the data used in the Oliveira et al. 2021 article, but I would presume it analysed the salival microbiomes of mainly North-American and Western European individuals. Can the difference in the taxon composition of your data also be the result of the different dietary culture of Japan and/or East Asia? I would assume that diet plays a substantial role in the formation of the general oral microbiome. I see that you elaborate on this a bit in Line 400-401, but I think a short paragraph could be also included about this after Line 328.

Response: We checked the origin of the data used in Oliveira et al. (2021) and found that they were confined to California. We agree with the Reviewer’s comment and added the following to the cited text: “In addition, because the previous meta-analysis was confined to salivary shotgun metagenome data obtained in California, the substantial difference in taxon composition may reflect differences in dietary culture between North America and Japan because diet plays a substantial role in the formation of the general oral microbiome.” (Lines 332–336)

Actually, your finding of these completely different genera compared to the previously reported “core salival microbiome” is much more interesting than your discussion of the Mogibacterium related results. Maybe you could elaborate on these findings in a subsequent article.

Response: Thank you for this constructive comment. We intend to elaborate on the findings in a subsequent article.
