# Supplementary material for: Shotgun metagenomic analysis of saliva microbiome suggests Mogibacterium as a factor associated with chronic bacterial osteomyelitis
Source: PLoS One. 2024 May 6;19(5):e0302569. doi: 10.1371/journal.pone.0302569 (PMC11073694; doi:10.1371/journal.pone.0302569)
Supplement: Attachment — Submitted filename: Answear to revisions.docx [file pone.0302569.s006.docx]

**Answer to revisions**

**Summary**

Thank you for considering my review suggestion. I think the presentation of your data improved significantly and the inclusion of the requested paragraph clarified the background workings of your analysis framework. The language and overall structure of your article is still very clear and straightforward.

In the next section, I would like to answer the answers to my reviews. Then I listed some suggestions concerning the Supplementary Tables. I wouldn’t like an answer for these I just included them as to make sure to really bring out the maximum from your excellent article.

**Answers**

The comparison also shows that the data points are the same between the 25th and 75th percentiles (shown as black dots in the middle boxes). Although the 75th percentile is indeed different (11.4 in Fig 1b and 8.5 in the figure created by reviewer’s R script), we confirmed that it is due to a difference in the quantile algorithm between JMP and R: the 75th percentile in Fig 1b (11.4) calculated by JMP is obtained using the 6th type quantile algorithm implemented in R, whereas that in the figure by the reviewer’s R script (8.5) is obtained using the 7th type quantile algorithm, which is the default in R.

Thank you for this thorough explanation I didn’t even know that quartile calculation was this complicated in R. I accept your answer.

According to your suggestion, we have added the following to the Methods: “According to previous studies [38, 39], we specified the total sum scaling (TSS) normalization (to ensure that the profile values ranged from 0 to 1) and arcsine square-root transformation (AST) (to stabilize the variance and improve parametric estimation models in the presence of violated data assumptions, such as normality and homoscedasticity) [37].” (Lines 216–219) We have also confirmed that the arcsine square-root transformation (AST) is conducted using the following R code "sign(x) * asin(sqrt(abs(x)))", and the taxonomic abundance of Mogibacterium (normalized to be from 0 to 1) before and after the transformation is distributed as shown below (left and right, respectively). The transformation seems to make the distribution less skewed.

Thank your for including this section in Methods. Your explanation clarifies the exact method that you were using, and thus I accept your answer. This helps to facilitate reproducibility furthermore provides opportunities for you to show your seemingly considerable experience working with this kind of data, which – I think – overall strengthens the viewers assessment of your ability to produce trustful results.

We appreciate your comment and agree with it. The illustration of the distribution above shows that the two samples at the right seem to be outliers, even after the arcsine square-root transformation (AST). Therefore, we added the following to the Discussion: “However, the sample size of the present study was small (N=5 in the chronic bacterial osteomyelitis group). Although all five patients with chronic bacterial osteomyelitis showed a greater taxonomic abundance of Mogibacterium than the five healthy controls, the abundance values for two of these patients appeared to be outliers that were only distinct relative to those of CNO patients (Fig 1b). Further studies should be performed with additional data on chronic bacterial osteomyelitis patients to explore the external validity of the study and determine the frequency of such outliers, which may represent a potential characteristic of chronic bacterial osteomyelitis. (Lines 390–397)

Thank you again, this section further strengthens the sincerity and transparency of your previous statements.

All together I think this article is appropriate for publication in this state.

**Suggestions**

The first row in Table_S1 contains 3 white spaces before the beginning of the sentence.

The name of the 5^th^ control individual is CN001_ with an ending underscore is this a typo?

The genus header in Table_S2 is still sampleID in the uploaded file but I presume you just didn’t upload the revised version along the Response to reviewers file.

I am sure the publisher has regulations in this regard, however I saw some really bad examples in even highly regarded publishers like Nature, that the name of the supplementary files were jumbled completely. Please name your files in a straightforward manner like Table_S1 or something like that because this really helps later reading and organisation for your readership.
